# Inflammation in delayed ischemia and functional outcomes after subarachnoid hemorrhage

**DOI:** 10.1186/s12974-019-1578-1

**Published:** 2019-11-11

**Authors:** Sung-Ho Ahn, Jude P. J. Savarraj, Kaushik Parsha, Georgene W. Hergenroeder, Tiffany R. Chang, Dong H. Kim, Ryan S. Kitagawa, Spiros L. Blackburn, H. Alex Choi

**Affiliations:** 1 Department of Neurology, University of Texas Health Science Center at Houston, Houston, TX USA; 2 Department of Neurology, Pusan National University School of Medicine, Research Institute for Convergence of Biomedical Science and Technology, Pusan National University Yangsan Hospital, Busan, South Korea; 3 Department of Neurosurgery, University of Texas Health Science Center at Houston, Houston, USA

**Keywords:** Subarachnoid hemorrhage, Delayed cerebral ischemia, Functional outcome, Inflammation, Cytokine network

## Abstract

**Background:**

Inflammatory mechanism has been implicated in delayed cerebral ischemia (DCI) and poor functional outcomes after subarachnoid hemorrhage (SAH). Identification of cytokine patterns associated with inflammation in acute SAH will provide insights into underlying biological processes of DCI and poor outcomes that may be amenable to interventions.

**Methods:**

Serum samples were collected from a prospective cohort of 60 patients with acute non-traumatic SAH at four time periods (< 24 h, 24–48 h, 3–5 days, and 6–8 days after SAH) and concentration levels of 41 cytokines were measured by multiplex immunoassay. Logistic regression analysis was used to identify cytokines associated with DCI and poor functional outcomes. Correlation networks were constructed to identify cytokine clusters.

**Results:**

Of the 60 patients enrolled in the study, 14 (23.3%) developed DCI and 16 (26.7%) had poor functional outcomes at 3 months. DCI was associated with increased levels of PDGF-ABBB and CCL5 and decreased levels of IP-10 and MIP-1α. Poor functional outcome was associated with increased levels of IL-6 and MCP-1α. Network analysis identified distinct cytokine clusters associated with DCI and functional outcomes.

**Conclusions:**

Serum cytokine patterns in early SAH are associated with poor functional outcomes and DCI. The significant cytokines primarily modulate the inflammatory response. This supports earlier SAH studies linking inflammation and poor outcomes. In particular, this study identifies novel cytokine patterns over time that may indicate impending DCI.

## Introduction

Aneurysmal subarachnoid hemorrhage (SAH) is a devastating disease caused by the sudden rupture of an intracranial aneurysm which is associated with a high risk of death or severe disability [1]. In those who survive the initial event, injury expansion after SAH can exacerbate underlying brain injury leading to secondary neurological injuries and poor functional outcomes [2, 3]. About 30% of SAH patients will develop delayed cerebral ischemia (DCI) [4, 5] which is a major complication that occurs at 3–14 days after SAH and can lead to poor outcomes [6, 7].

Recently, inflammation after SAH has received attention, mainly as a mediator of injury expansion and as a potential target for therapies, to improve outcomes [8–10]. SAH induces a peripheral immune response and activated peripheral immune cells are recruited to the brain parenchyma [11] where they release cytokines [12] that induce the upregulation of intrinsic receptors leading to widespread inflammation [13]. Systematic inflammatory activity peaks at 24–48 h after SAH and correlates with poor clinical status at admission [14]. Also, previous studies investigating biomarkers specific to DCI have identified several pro-inflammatory cytokines including TNF-a, IL-1, and IL-6 and anti-inflammatory cytokine including IL-10 and several growth factors [15] as well. Systemic inflammation has been proposed as one factor that increases susceptibility to DCI after SAH [16].

In this study, we aim to identify biomarkers and characterize the immune response at different stages after SAH using a combination of statistical and network informatics techniques. We hypothesize that DCI and poor functional outcomes are associated with an increased systemic inflammatory response. The characterization of the inflammatory response can lead to a better understanding of the pathophysiology after SAH.

## Methods

### Study population

Patients with acute SAH admitted to the Neuroscience Intensive Care Unit at the Memorial Herman Hospital-Texas Medical Center, Houston, TX, between July 2013 and March 2015 were enrolled in a prospective observational study. Institutional review board approval was obtained (HSC-MS-12-0637). Inclusion criteria were (a) age > 18 years and (b) spontaneous aneurysmal SAH diagnosed by computed tomography (CT) within 24 h of ictus or by xanthochromia in cerebrospinal fluid if the CT was not diagnostic. Exclusion criteria were (a) non-aneurysmal SAH due to trauma, arteriovenous malformation, or mycotic aneurysms and (b) comorbidities that could significantly affect baseline inflammation including autoimmune diseases, suspicion of infection, history of malignancy, and current pregnancy. Written informed consent was obtained from the patient or surrogate.

### Clinical and imaging data and definitions

Clinical data collected included age, sex, medical and social history, neurological status at admission based on the World Federation of Neurosurgical Societies scale (WFNS grade) [17], and loss of consciousness at ictus. Admission CT images were adjudicated for the presence of intraventricular hemorrhage, hydrocephalus, and cerebral infarction [18, 19]. All CT scans were independently evaluated by a study neurologist for the amount and location of blood as categorized by the modified Fisher score (mFS) [20, 21].

### Outcome data definitions

We adhered to the proposed definition of DCI [22] as “The occurrence of focal neurological impairment (such as hemiparesis, aphasia, apraxia, hemianopia, or neglect), or a decrease of at least 2 points on the Glasgow Coma Scale (either on the total score or on one of its individual components [eye, motor on either side, verbal]). This should last for at least 1 hour, is not apparent immediately after aneurysm occlusion, and cannot be attributed to other causes by means of clinical assessment, CT or MRI scanning of the brain, and appropriate laboratory studies.” DCI was diagnosed only after rigorous exclusion of other possible causes. Delayed cerebral ischemia was adjudicated through consensus of at least two attending neurointensivists in weekly research meetings. In the rare instance when no consensus can be achieved, the principal investigator made the final determination. Characteristics of the CT scan including IVH, modified Fisher scale, hydrocephalus, and cerebral infarction were graded by the PI of the study blinded to other aspects of the clinical course. Specifically, any blood in the ventricles was graded as IVH. Functional outcome was assessed by the 0–6 modified Rankin Scale (mRS). The mRS at discharge was assessed by the attending neurointensivist taking care of the patient. All physicians are certified to grade the mRS. The 3-month mRS was obtained via a follow-up phone call by a trained research personnel using a standardized questionnaire. Unfavorable outcome at 3 months after discharge was defined as a mRS of 3–6. Subjects were treated according to standard guidelines.

### Sample collection and processing protocol

Serum samples were collected at four predetermined time points after admission: < 24 h (*T*_1_), between 24 and 48 h (*T*_2_), 3–5 days (*T*_3_), and 6–8 days (*T*_4_). Forty-one serum cytokine concentrations in picograms per milliliter were measured following the manufacturer’s protocol using a MAGPIX magnetic bead-based ELISA 41-plex assay (EMD Millipore, Billerica, MA): sCD40L, EGF, Eotaxin (CCL11), FGF-2, Flt-3 ligand (FLT3L), Fractalkine (CX3CL1), G-CSF (CSF3), GM-CSF (CSF2), GRO (CXCL1), IFN-α2, IFN-γ, IL-1α, IL-1β, IL-1ra, IL-2, IL-3, IL-4, IL-5, IL-6, IL-7, IL-8, IL-9, IL-10, IL-12 (p40), IL-12 (p70), IL-13, IL-15, IL-17A, IP-10, MCP-1 (CCL2), MCP-3 (CCL7), MDC (CCL22), MIP-1α, MIP-1β, PDGF-AA, PDGF-AB/BB, CCL5, TGF-α, TNF-α, TNF-β, and VEGF. They were analyzed based on previously published protocols [14] (see Additional file 3 under *Cytokine analysis*).

### Statistical analysis

Study subjects’ characteristics were described using unpaired Student’s *t* tests for continuous variables and chi-square or Fisher’s exact tests for categorical variables. The non-parametric Mann-Whitney *U* test (*p* <  0.10) was used to identify candidate cytokines in each time period that differentiated DCI and functional outcomes (Additional file 3: Table S1). Using these candidate cytokines, multivariate logistic regression analyses were performed to find cytokines independently associated with DCI and unfavorable functional outcome after adjusting for clinically relevant variables including age, sex, mFS (as a radiographic surrogate for injury severity), and WFNS grade (as a clinical surrogate for injury severity), respectively. The results of the multivariate logistic regression analysis were reported as an odds ratio (OR) at a 95% confidence interval (CI). A *p*-value of ≤ 0.05 was considered statistically significant (two-tailed). All statistical analyses were performed using SPSS 17.0 (SPSS Inc., Chicago, IL).

Two-dimensional correlation network graphs were constructed to show linked relationships among cytokines and to identify clusters of cytokines associated with DCI or poor outcomes at specific time periods. In these correlation networks, each cytokine was represented as a node (vertex) and the relationship between two cytokines as an edge (undirected line). Cytokine measurements (picograms per milliliter) were normalized via Box-Cox transformations and Pearson’s correlation coefficient (PCC) was calculated (R v3.1.3) for each pair of the 41 cytokines to represent the “strength” of the edge connecting the two cytokines. Only strong edges with a PCC of at least 0.75 (and *p* <  0.001) were used to construct correlation network graphs for DCI/no-DCI and good/poor functional outcome groups (Cytoscape v3.2.1).

## Results

### Demographics

During the study period, 151 patients with an acute aneurysmal SAH were admitted to our unit. One hundred twelve patients met the inclusion criteria and consented to participate in the study. Since the baseline inflammatory status (and cytokine levels) can vary subject-to-subject, to preserve homogeneity, we prioritized the inclusion of consecutive subjects from whom the maximum number of samples were available from *T*_1_ to *T*_4_. Of the 60 subjects included, 52 subjects had 3 or more samples (a total of 189 samples) and only 8 subjects had less than 3 samples (a total of 14 samples). We analyzed 203 samples in total. The differences in demographics between subjects who were included in the study versus subjects who were not included are shown (Additional file 3: Table S2). The mean age of the subjects was 52.2 ± 13.0 years (range 23–83 years) and 46 (76.7%) were women. Seventeen subjects had their aneurysm clipped, 40 had embolization with coils, and 3 subjects were untreated. Fourteen subjects (23.3%) developed DCI on an average of 6.2 days after admission. Sixteen (26.7%) subjects were reported to have poor functional outcome (mRS ranging from 3 to 6 score at 3 months).

### Clinical variables across groups

There were no significant differences in clinical variables between the DCI and no-DCI group. Subjects with poor functional outcomes were older and had higher WFNS grade and LOC at ictus, higher mFS grade, and hydrocephalus on admission CT compared to those with good functional outcomes (Table 1). In the multivariable model, age and high-grade WFNS were independent predictors of poor functional outcome (Additional file 3: Table S3).

**Table 1:** Clinical variables across DCI and functional outcomes

Variable	DCI		*p* value^†^	Functional outcomes at 3 months		*p* value^†^
	DCI (*n* = 14)	no-DCI (*n* = 46)		Poor (*n* = 16)	Good (*n* = 44)	
Age (years ± SD)	52.4 ± 12.1	52.2 ± 13.4	0.96	60.7 ± 13.0	49.1 ± 11.8	*< 0.01*
Sex, male	1 (7.1)	13 (28.3)	0.15	5 (31.3)	9 (20.5)	0.38
Risk factors						
Hypertension	8 (57.1)	29 (63.0)	0.69	12 (75.0)	25 (56.8)	0.20
Diabetes mellitus	1 (7.1)	6 (13.0)	1.00	1 (6.3)	6 (13.6)	0.66
Current smoking	5 (35.7)	21 (45.7)	0.51	6 (37.5)	20 (45.5)	0.58
Clinical exam on admission						
WFNS grade	2 [1–4]	2 [1–4]	0.71	4 [2.5–4.5]	1 [1–2]	*< 0.01*
LOC at ictus	5 (35.1)	18 (39.1)	0.82	10 (62.5)	13 (29.5)	*0.02*
Radiographic findings						
Modified Fisher grade	3 [3–3]	3 [3–3]	0.90	3 [3–4]	3 [3–3]	*0.01*
IVH	7 (50.0)	27 (58.1)	0.57	11 (68.8)	23 (52.3)	0.26
Hydrocephalus	8 (57.1)	28 (60.9)	0.80	13 (81.3)	23 (52.3)	*0.04*
Therapeutic intervention						
Coiling	9 (64.3)	31 (67.4)	0.83	7 (77.8)	33 (64.7)	0.44

*mRS* modified Rankin Scale, *WFNS* World Federation of Neurological Surgeons, *DCI* delayed cerebral ischemia, *LOC* loss of consciousness, *IVH* intraventricular hemorrhage, *DCI* delayed cerebral ischemia

Variables are presented as mean ± SD, median [interquartile range], or number (%)

^†^*p* values are calculated by Pearson’s chi-square test or Fisher’s exact test, or Student’s *t* test as appropriate

### Cytokines associated with DCI and poor functional outcome

At *T*_1_, *T*_2_, *T*_3_, and *T*_4_, the levels of 2, 4, 3, and 13 cytokines, respectively, were significantly different across the DCI and no-DCI group. At the same time points, the levels of 8, 7, 5, and 11 cytokines, respectively, were significantly different across the good and poor outcome groups (Additional file 3: Table S1). Multivariable logistic regression analyses reveal that higher PDGF-ABBB at *T*_2_, higher CCL5 at *T*_2_ and *T*_3_, lower IP-10 at *T*_1_, and lower MIP-1α at *T*_4_ was independently associated with DCI after adjusting for age, sex, mFS, and WFNS. In addition, IL-6 at *T*_2_ and MCP-1 at *T*_4_ were independently associated with poor mRS at 3 months after adjusting for age, sex, and mFS but not WFNS grade (Table 2).

**Table 2:** Adjusted odds ratio of cytokines for prediction of DCI and poor functional outcome

Variables	Multivariable^a^		Multivariable^b^		Multivariable^c^	
	OR	95% CI	OR	95% CI	OR	95% CI
DCI						
IP-10^d^ (T_1_), per 10 pg/mL increase	0.96	0.918–0.998	0.96	0.916–0.997	0.95	0.910–0.995
PDGF-ABBB^d^ (T_2_), per 10 pg/mL increase	1.02	1.004–1.029	1.02	1.004–1.029	1.02	1.005–1.030
CCL5 (T_2_)^d^, per 10 pg/mL increase	1.01	1.003–1.019	1.01	1.003–1.019	1.01	1.003–1.019
CCL5^d^ (T_3_), per 10 pg/mL increase	1.01	1.003–1.022	1.01	1.002–1.022	1.01	1.002–1.023
MIP-1α^d^ (T_4_), per 1 pg/mL increase	0.65	0.443–0.943	0.65	0.447–0.950	0.64	0.421–0.958
Poor clinical outcome at 3 months						
IL-6 (T_2_)^d^, per 1 pg/mL increase	1.13	1.020–1.262	1.13	1.018–1.257	1.04	0.940–1.152
MCP-1 (T_4_)^d^, per 10 pg/mL increase	1.05	1.009–1.098	1.04	1.001–1.089	1.05	0.992–1.108

*OR* odds ratio, *CI* confidence interval

^a^Adjustments were made for age and sex

^b^Adjustments were made for age, sex, and mFS

^c^Adjustments were made for age, sex, mFS, and WFNS grade

^d^Odds ratio of an individual cytokine after adjusting main covariables

**Fig. 1:**
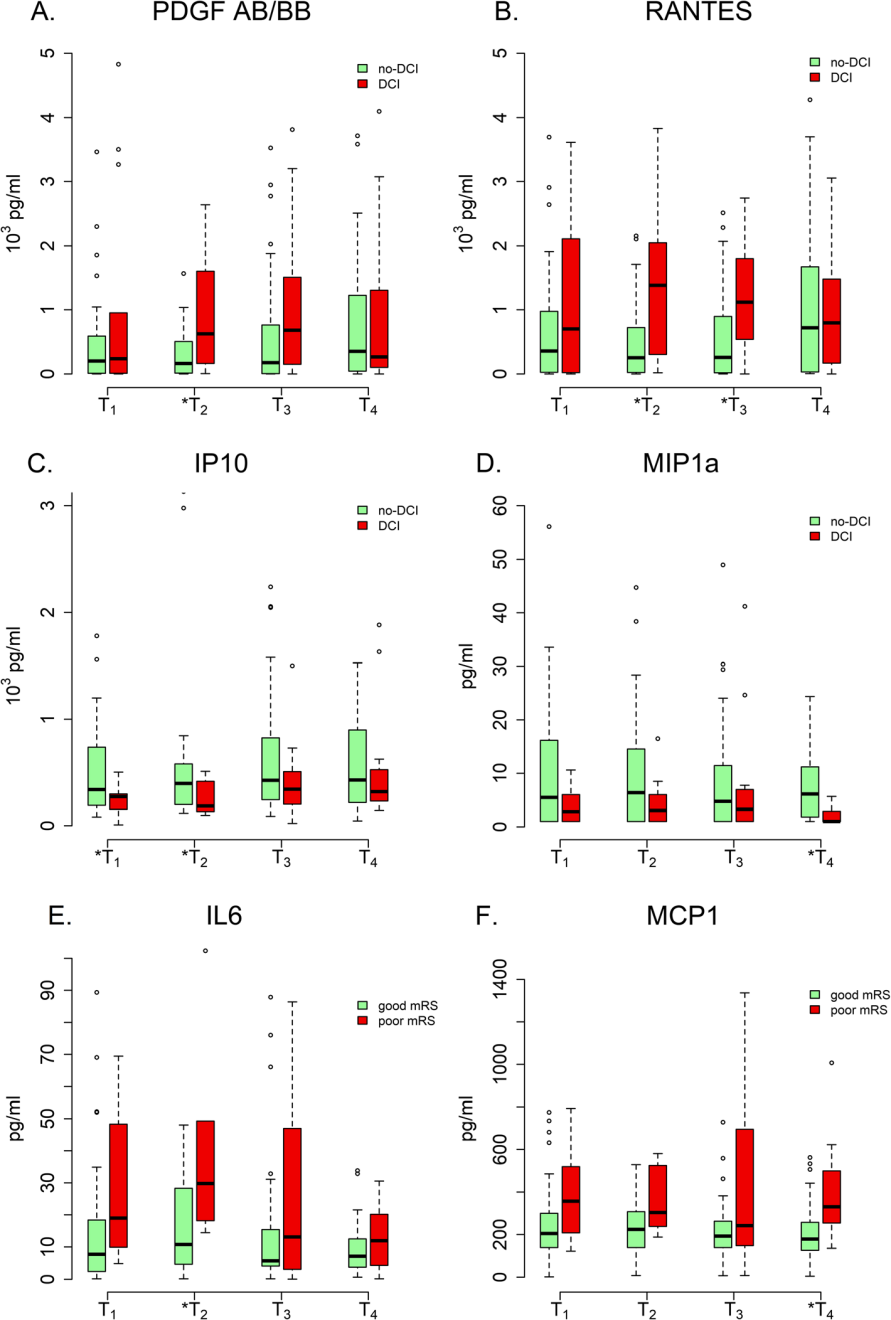
**a**–**f** Changes in median levels of cytokines across DCI and functional outcomes. Asterisk indicates significance of the cytokine (*p* < 0.05) in predicting DCI (after adjustment for age, sex, and mFS grade) and poor functional outcome at 3-months (after adjustment for age, sex, mFS, and WFNS grade)

We plotted the trends of median values of six cytokines (PDGF-ABBB, RANES, IP-10, MIP-1α, IL-6, and MCP-1) that were independently associated with either DCI or poor outcome. Median levels of PDGF-ABBB (particularly at *T*_2_) and CCL5 (particularly at *T*_2_ and *T*_3_) were consistently higher, and median levels of IP-10 (particularly at *T*_1_ and *T*_2_) and MIP-1α (particularly at *T*_4_) were consistently lower in the DCI group compared to the no-DCI group (Fig. 1a–d). Median levels of IL-6 (particularly at *T*_2_) and MCP-1 (particularly at *T*_4_) were consistently higher in subjects with poor functional outcome than those with good functional outcome (Fig. 1e, f).

### Network analysis in DCI and functional outcomes

**Fig. 2:**
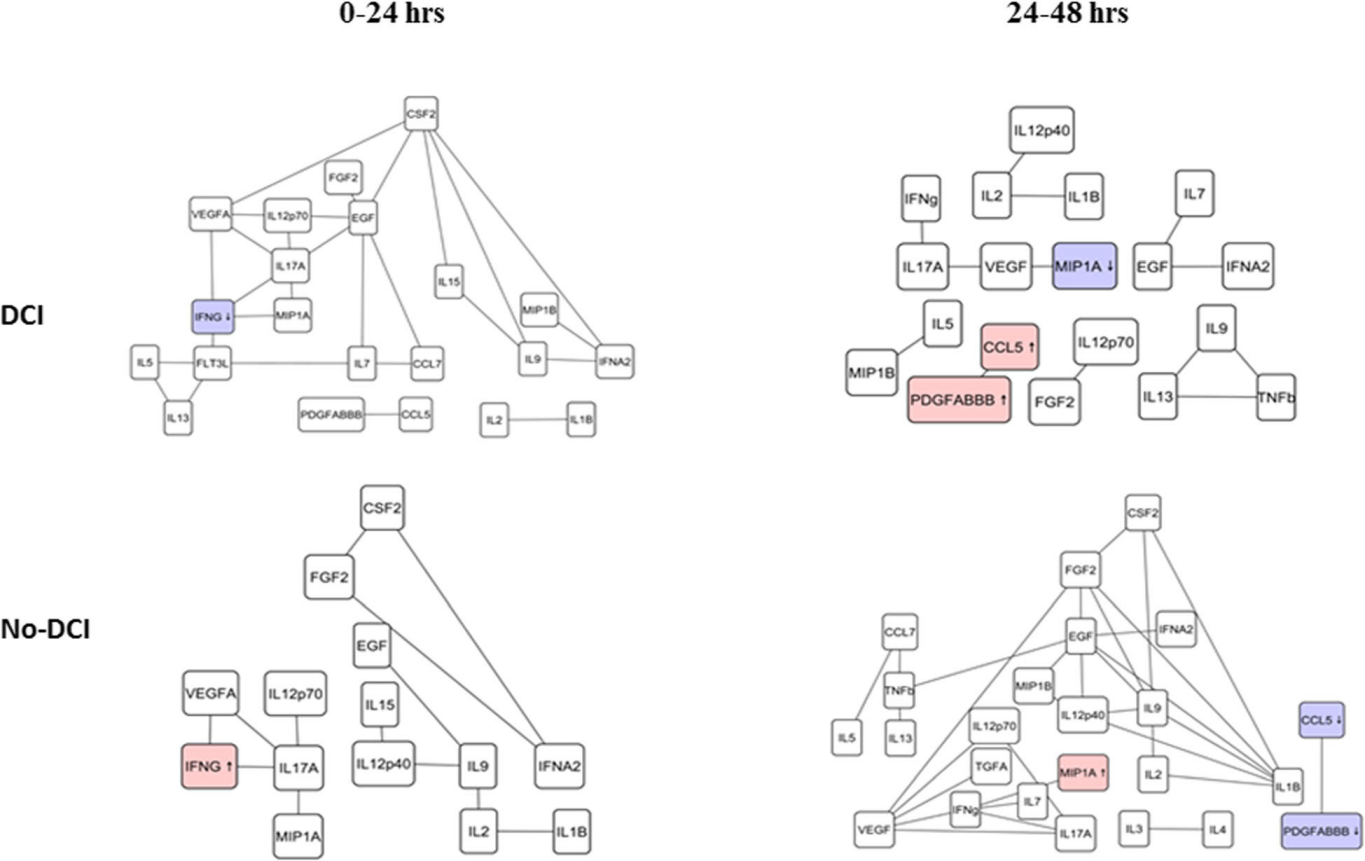
Dynamics of inflammatory mechanism across DCI and no-DCI cohort within 48 h. Two large correlated clusters, one including VEGFa, EGF, and FGF2 and another consisting of IL9, IL15, and IFNA2 were observed in the DCI group (left). These clusters were minimally connected in the no-DCI cohort. Upwards arrow indicates elevated concentration levels of the cytokine in the DCI group compared to the no-DCI group. Downwards arrow indicates decreased concentration levels of the cytokine in the no-DCI group compared to the DCI group

We identified distinct cytokine associations in the DCI and functional outcome group from *T*_1_ to *T*_4_. In the DCI network at *T*_1_ and *T*_2_, we observed a cluster including VEGFA, EGF, and FGF2 and another cluster consisting of IL9, IL15, and IFNA2 in the DCI cohort. Another small cluster including the cytokines PDGF-ABBB and CCL5 was common to both groups and the cytokines were elevated and independently associated with DCI. This cluster was observed from *T*_2_ to *T*_4_ as well (Fig. 2 and Additional file 1: Figure S1).

**Fig. 3:**
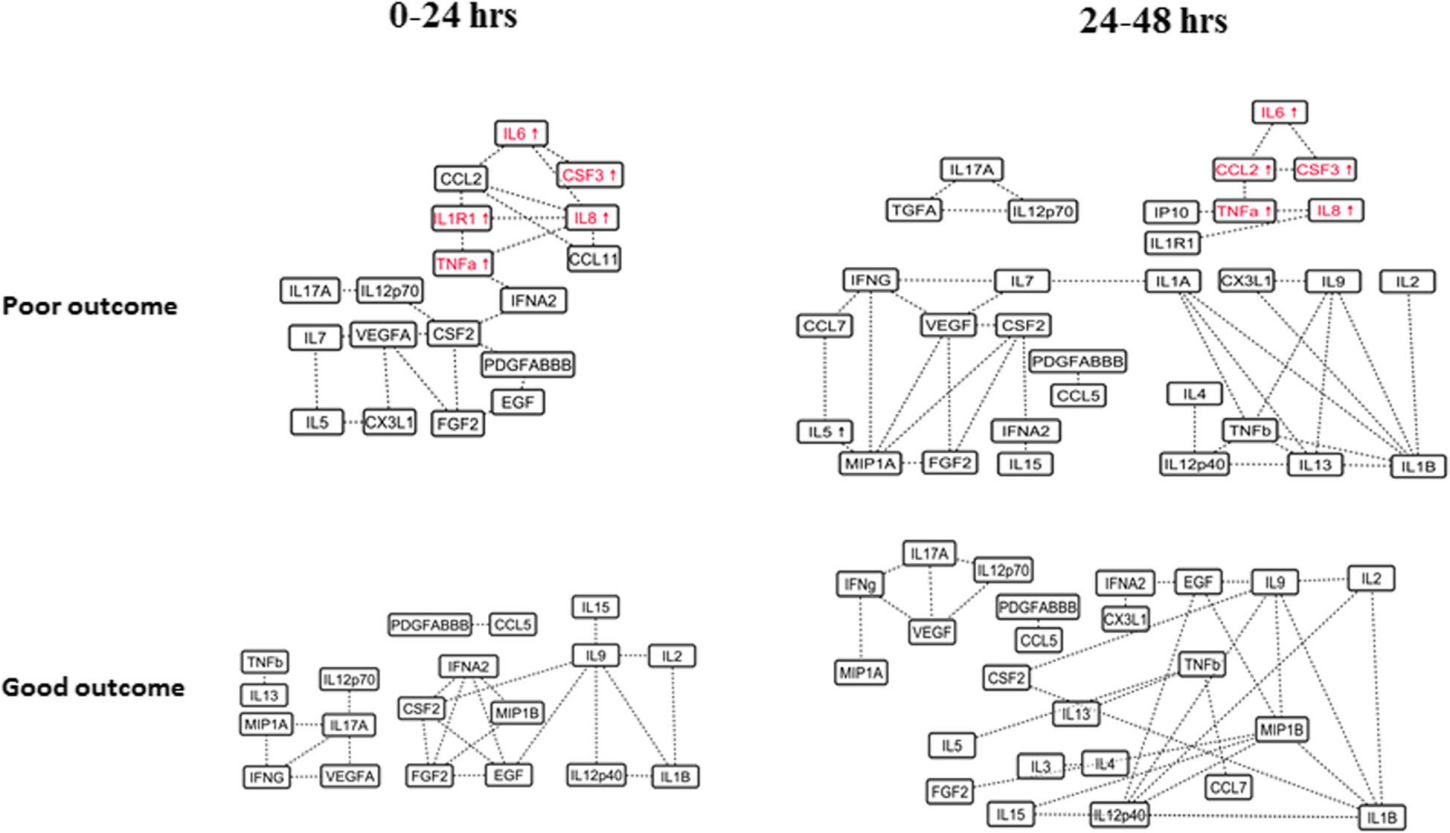
Dynamics of inflammatory mechanisms across the functional outcome groups within 48 h. A cytokine cluster comprising of IL1R1, TNFa, IL8, IL6, CCL2 (MCP1), CCL11, and GCSF (CSF3) was observed in the poor outcome group. Upwards arrow indicates elevated concentration levels of the cytokine in the poor functional outcome compared to the good functional outcome. This cluster was observed at *T*_1_ and *T*_2_ and persisted over time

For functional outcome networks, IL-6 and MCP-1 (CCL2) were highly correlated (which were independently associated with poor outcomes) and extend to other cytokines. IL-6 was closely associated with a group of cytokines (including CCL2, TNF-α, IL-8, IL-10, IL-1Ra, and IP-10) which formed a unique cluster in poor outcome group from *T*_1_ to *T*_4_ (Fig. 3 and Additional file 2: Figure S2).

## Discussion

We investigated the peripheral immune response associated with DCI and functional outcomes. We measured the levels of several serum cytokines at different acute time points after SAH and described them using statistical and informatics methods. In subjects who developed DCI, platelet-released cytokines including PDGFs and CCL5 were elevated and IP-10 and MIP-1α were lower. Early elevations of IL-6 and MCP-1 in post-SAH were independently associated with poor functional outcomes at 3 months. Furthermore, network analysis reveals presence of distinct cytokine clusters across the groups.

### Peripheral immune response and DCI

PDGF-ABBB (at *T*_2_), CCL5 (at *T*_2_ and *T*_3_), IP-10 (at *T*_1_ and *T*_2_), and MIP-1α (at *T*_4_) were independently associated with DCI. Platelet-released PDGF is a mitogenic growth factor linked to peripheral thrombosis, and increased PDGF levels have been reported in ischemic brain injury and brain puncture wounds [23]. CCL5 is a member of the CC-chemokine family produced by a variety of cells, including T lymphocytes, platelets, endothelial cells, smooth muscle cells, and glial cells. It can interact with chemokine receptors that recruit leukocytes to inflammatory sites, helping migration of leukocytes across the endothelium contributing to the pathogenic process of arterial injury and atherosclerosis [24, 25]. T-cell recruitment and activation in the injured brain parenchyma could be the prelude to secondary ischemic injury [26, 27] and have been shown to migrate across the vessel wall in response to brain injury [28]. T cell-directed chemokines (including CCL5) are particularly important in stroke prediction including DCI [29]. Interestingly, IP-10 and MIP-1α were lower in DCI compared to the no-DCI group. Both cytokines have shown to be involved in both pro-inflammatory and regulatory mechanisms. IP-10 signaling, with the combination of CCL5, is a known promoter of T cell recruitment and activation [30]. Two known pathways involving IP-10, the restraining of IP-10 production by CCL5 [31] and the inhibition of regulatory T cell recruitment by IP-10 [30], have been previously implicated in controlling inflammatory response and in neuroprotection after ischemic insult [26, 27]. MIP-1α is primarily involved in inflammation and homeostasis [32] and plays an important role in both acute and chronic inflammation, primarily in the role of recruiting pro-inflammatory cells. It has been implicated in the development of ischemic brain injury [15]; however, they also promote healing by inducing inflammatory responses against external injuries [32].

### Cytokine clusters in DCI

A small cluster (including platelet-released PDGF-ABBB and CCL5) was observed in the DCI and no-DCI group. The levels of most cytokines in this cluster were significantly different across the DCI/no-DCI group at *T*_2_ and *T*_3_ suggesting increased coagulatory and inflammatory activity prior to DCI (which typically occurs after *T*_3_). Previous studies have shown that early activation of the coagulation cascade and impairment of the fibrinolytic cascade can lead to DCI after SAH [33] and the levels of platelet-activating factors increase within 4 days after SAH [34]. Elevated concentrations of the von Willebrand factor (the primary initiator of coagulation) have been shown to be associated with DCI as well [35, 36]. Furthermore, other studies have shown that overactive inhibition of fibrinolysis is associated with DCI [37]. Another cluster including IFNg, MIP1A, IL17a, IL12p70, and VEGFa was observed in DCI. VEGF is involved in neurogenesis and in the inhibition of apoptosis [38], promoting neuroprotection. Also, vascular CD4+ TH1 cells induce the release of IL12 and IFNg [39] which are mediators of tissue damage.

In addition to cytokine clusters, we observed quantitative differences in the networks as a whole. At *T*_2_ (typically a period prior to when DCI occurs), the DCI and no-DCI group had a similar number of cytokines and significant correlations (19 and 13 vs 24 and 32). At *T*_4_ (a period where DCI typically occurs), the DCI group had a higher number of cytokines and correlations compared to no-DCI (22 and 14 vs 12 and 10) suggesting an overall increase in systematic inflammatory mechanisms. Additionally, the concentrations of 13 cytokines differed significantly across the DCI and no-DCI groups at *T*_4_ (more so than in any other time period) suggesting a peak in systemic inflammation at *T*_4_. Overall, there is a simultaneous activation of angiogenesis, inflammation, and thrombotic mechanisms during the course of DCI [9].

### Peripheral immune response and functional outcomes

IL-6 (at *T*_2_) and MCP-1 (at *T*_4_) were independently associated with functional outcomes confirming the importance of the association between early inflammation and clinical outcomes. Our findings are consistent with previous studies where IL-6 and MCP-1 were shown to be sequentially expressed in CSF after SAH [40, 41]. MCP-1 is a CC chemokine family member and can regulate leukocyte trafficking by affecting their chemotactic activity, activating inflammatory cells, and modulating interactions between leukocytes and endothelial cells [42]. Previous studies have shown that activated mononuclear leukocytes can be found surrounding the major cerebral arteries in the subarachnoid space after SAH [43], and MCP-1 has been found to be significantly expressed in the major cerebral arteries in experimental vasospasm [7]. Our findings confirm that early rise in serum IL-6 and MCP-1 are associated with poor clinical status and consequently poor clinical outcomes. This finding further emphasizes the importance of “early brain injury” (defined as the injury process occurring < 72 h after aneurysm rupture) and its impact on functional outcomes [44, 45].

### Cytokine clusters in poor functional outcomes

In the poor functional outcome group, a unique cluster including the cytokines IL1R1, TNFa, IL8, IL6, CCL2, CCL11, IP10, and CSF3 (Fig. 3) was observed at *T*_1_ to *T*_4_. This suggests the presence of inflammatory mechanisms mediated by IL1a, TNFa, and IL6. IL1 has been shown to upregulate the pro-inflammatory IL6. It is released by macrophages and microglia via an endogenous DAMP-mediated inflammatory response triggered by heme released from lysed blood cells [46]. IL1R1 is a IL1 receptor antagonist which blocks the actions of IL1 and has been identified as a therapeutic candidate in ischemic diseases [47]. In pre-clinical models, administration of IL1RA has been shown to reduce blood-brain barrier disruption and mitigation of cell death, and, clinically, it has also shown to reduce inflammation as well [48]. We observed a high correlation between TNFa and IL8 in MRS ≥ 4 group in comparison to MRS ≤ 3 (0.80 vs 0.33 and *T*_1_). This strong correlation persisted through *T*_4_ indicating the mediation of inflammation via persistent activation of the NF-kB-dependent pathway. It is likely that inflammation-triggered oxidative stress increases TNFa, which in turn increases IL8 expression [49]. TNFa activates vascular Rac-1 promoting vasoconstriction, and targeting TNFa has shown to mitigate neuronal injury [50, 51]. Network analysis shows strong correlation between TNFa, IL8, and IL1R1 suggesting that IL1- and NF-kB-mediated responses are associated. Additionally, a strong correlation between IL-6 and MCP-1 was observed in the poor outcome group. It is purported that IL-6 may induce MCP-1 expression via signal transducer and activator of transcription 3 after SAH [15]. Overall, network analysis suggests simultaneous activation of pro-inflammatory and anti-inflammatory mechanisms sustained through the acute stages of SAH which consequently leads to poor functional outcomes. This analysis also shows that cytokines that are not significantly elevated across groups can still play an important role in the underlying inflammatory mechanisms.

## Limitation

This study is a single-center observational study and has a relatively small number of enrolled subjects. This can be a possible source of confounders and bias. Second, while several methodological efforts were employed to remove weak correlations, the complete elimination of spurious correlations in the network analysis is not possible. Third, while we excluded subjects with conditions (including suspicion of infection) at admission, a modest number of subjects developed infections (mostly in the *T*_3_ and *T*_4_ period). Infections are an issue that involves patient cohorts admitted in the ICU; however, we believe that they have a negligible effect on the inflammatory status on subjects in our cohort (see Additional file 3 under *Infection).* Fourth, as per institutional protocol, all patients are administered steroids on admission. Patients were started on dexamethasone 4 mg q6 on presentation and tapered over a week. There is considerable variation in this across centers; unfortunately, we cannot comment on this as all our patients receive steroids. However, since the subjects across both groups (DCI vs no-DCI and good vs poor outcomes) received steroids, it is likely that the inflammatory findings are consequential of SAH pathophysiology. Fifth, the nature of the study design only permits the observation of associations between the cytokines and does not present any causal mechanisms. To ascertain whether the observations contribute to DCI or poor outcomes, interventional trials are to be undertaken. Fifth, only peripheral inflammation was examined. Examining central inflammation by analyzing cerebrospinal fluid and/or cerebral microdialysis analysis could further our understanding of the underlying pathophysiological mechanisms.

## Conclusion

We identified serum cytokines at different time points that were independently associated with DCI and functional outcomes. The disruption in immune activity at the acute phase of SAH persisted through the subacute phase as evidenced by the complex cytokine interactions captured through network analysis. This study is an important step towards an integrated approach to describe global inflammatory reaction after SAH.

## Supplementary information

**Additional file 1:** **Figure S1.** Dynamics of inflammatory mechanims across DCI beyond 48 hours. A cluster including VEGF, FGF2, IL12p70, IL17A and IFNg, and separate small cluster involving PDGF-ABBB and CCL5 was observed. Quantitative difference in serum level of these cytokines was further increased especially at 6–8 days after stroke onset. ‘↑’ indicates elevated concentration levels of the cytokine in the DCI group compared to the no-DCI group. ‘↓’ indicates decreased concentration levels of the cytokine in the no-DCI group compared to the DCI group. (TIF 98 kb)

**Additional file 2: Figure S2.** Dynamics of inflammatory mechanisms across poor functional outcomes beyond 48 hours. The cluster comprising of IL1R1, TNFa, IL8, IL6, CCL2 (MCP1), CCL11 and GCSF (CSF3), IL10 and IP10 was observed in the poor outcome group at the acute stages after SAH. (TIF 126 kb)

**Additional file 3: Table S1.** Cytokines associated with DCI and functional outcomes in each time point after SAH. **Table S2.** Differences in baseline variables between subjects who were enrolled and not-enrolled in the study. **Table S3.** Multivariable logistic regression analysis to identify predictors of poor clinical outcomes. (DOCX 54 kb)

## Data Availability

The datasets used and/or analyzed during the current study are available from the corresponding author on reasonable request.

## Abbreviations

CT: computed tomography
DCI: Delayed cerebral ischemia
mFS: Modified Fisher score
mRS: Modified Rankin Scale
OR: Odds ratio
SAH: Subarachnoid hemorrhage
WFNS: World Federation of Neurosurgical Societies scale
