# Supplementary figures and images for: Inflammation in delayed ischemia and functional outcomes after subarachnoid hemorrhage
Source: J Neuroinflammation. 2019 Nov 11;16:213. doi: 10.1186/s12974-019-1578-1 (PMC6849179; doi:10.1186/s12974-019-1578-1)

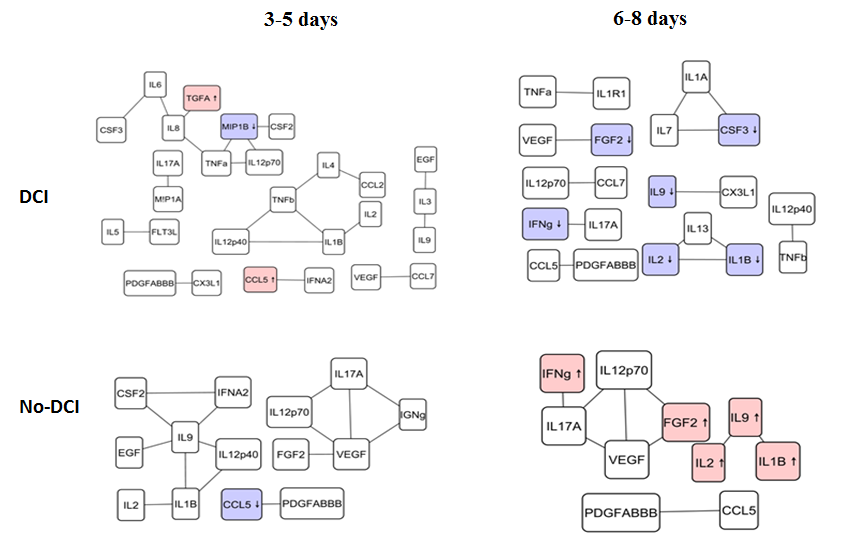

Supplement: Supplementary file 1 — Additional file 1: Figure S1. Dynamics of inflammatory mechanims across DCI beyond 48 hours. A cluster including VEGF, FGF2, IL12p70, IL17A and IFNg, and separate small cluster involving PDGF-ABBB and CCL5 was observed. Quantitative difference in serum level of these cytokines was further increased especially at 6–8 days after stroke onset. ‘↑’ indicates elevated concentration levels of the cytokine in the DCI group compared to the no-DCI group. ‘↓’ indicates decreased concentration levels of the cytokine in the no-DCI group compared to the DCI group. (TIF 98 kb) [file 12974_2019_1578_MOESM1_ESM.tif]

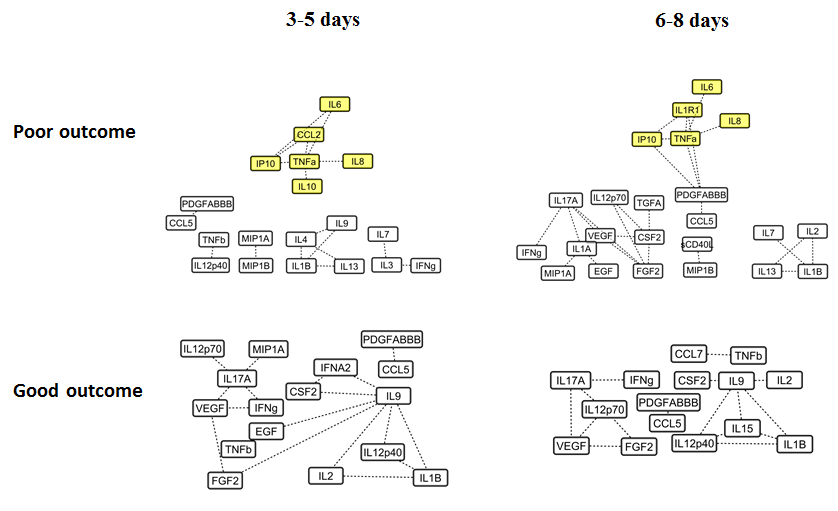

Supplement: Supplementary file 2 — Additional file 2: Figure S2. Dynamics of inflammatory mechanisms across poor functional outcomes beyond 48 hours. The cluster comprising of IL1R1, TNFa, IL8, IL6, CCL2 (MCP1), CCL11 and GCSF (CSF3), IL10 and IP10 was observed in the poor outcome group at the acute stages after SAH. (TIF 126 kb) [file 12974_2019_1578_MOESM2_ESM.tif]
