# Supplementary material for: Inflammation in delayed ischemia and functional outcomes after subarachnoid hemorrhage
Source: J Neuroinflammation. 2019 Nov 11;16:213. doi: 10.1186/s12974-019-1578-1 (PMC6849179; doi:10.1186/s12974-019-1578-1)
Supplement: Supplementary file 3 — Additional file 3: Table S1. Cytokines associated with DCI and functional outcomes in each time point after SAH. Table S2. Differences in baseline variables between subjects who were enrolled and not-enrolled in the study. Table S3. Multivariable logistic regression analysis to identify predictors of poor clinical outcomes. (DOCX 54 kb) [file 12974_2019_1578_MOESM3_ESM.docx]

**Table S1. Cytokines associated with DCI and functional outcomes in each time point after SAH**

|  | DCI until discharge | | | |  | mRS grade at 3 months | | | |
| --- | --- | --- | --- | --- | --- | --- | --- | --- | --- |
|  | DCI+ (vs DCI-) | | | |  | Poor mRS (vs Good mRS) | | | |
| Cytokines | T_1_  14 (vs 45) | T_2_  12 (vs 29) | T_3_  12 (vs 40) | T_4_  11 (vs 39) |  | T_1_  16 (vs 43) | T_2_  10 (vs 31) | T_3_  13 (vs 39) | T_4_  14 (vs 36) |
| MIP-1α | - | L | - | L* |  | - | - | - | - |
| MIP-1β | - | - | L* | L* |  | - | - | - | - |
| CCL5 | - | H* | H* | - |  | - | - | - | - |
| IFN-γ | L | - | - | L* |  | - | - | - | - |
| TGF-α |  |  | H | L |  | - | - | - | L |
| IP-10 | L* | L* | - | - |  | H* | - | H* | H* |
| GCSF | - | - | - | L* |  | H | H | H* | - |
| MCP-1 | - | - | - | H |  | H* | H* | - | H* |
| TNF-α | - | - | - | - |  | H* | H* | - | H |
| IL-6 | - | - | - | - |  | H* | H* | - | - |
| IL-8 | - | - | - | - |  | H* | H* | H* | - |
| IL-10 | - | - | - | - |  | H* | H | H | H* |
| PDGF- ABBB | - | H* | - | - |  | - | - | - | - |
| PDGF-AA | - | - | - | - |  | - | - | - | L* |
| FGF-2 | - | - | - | L |  | - | - | - | L |
| IFN-α2 | - | - | - | L |  | - | - | - | L* |
| GM-CSF | - | - |  | L |  | - | - | - | L* |
| sCD40L | - | - | - | - |  | H | - | - | - |
| IL-1β | - | - | - | L |  | - | - | - | - |
| IL-1Ra | - | - | - | - |  | - | - | H | - |
| IL-2 | - | - | - | L* |  | - | - | - | - |
| IL-5 | - | - | - | - |  | - | H* | - | - |
| IL-9 | - | - | - | L* |  | - | - | - | H* |
| MCP-3 | - | - | - | L* |  | - | - | - | L* |
| **Total ^a^** | **2** | **4** | **3** | **13** |  | **8** | **7** | **5** | **11** |

DCI = delayed cerebral ischemia; mRS = modified Rankin scale; L = low level; H = high level

**^a^**Number of cytokine which showed either high (H) or low (L) level of cytokine in subjects with DCI or poor functional outcome (versus those without DCI or good functional outcome) with a *p*-value < 0.10 by Mann-Whitney U test for non-parametric test in each time point were listed.*Statistically significant difference in cytokine level with a *p*-value < 0.05 by Mann-Whitney U test for non-parametric test among candidate cytokine which showed either high (H) or low (L) level of cytokine according to the DCI or functional outcome.

**Table S2. Differences in baseline variables between subjects who were enrolled and not-enrolled in the study**

|  | Eligible subjects | |  |
| --- | --- | --- | --- |
| Variable | enrolled  (N = 60) | not-enrolled  (N = 52) | *p-*value† |
| Age (years) | 52.2 ± 13.0 | 56.5 ± 13.0 | 0.09 |
| Sex, male | 14 (23.3) | 20 (38.5) | 0.08 |
| **Risk factors** |  |  |  |
| Hypertension | 37 (61.7) | 28 (53.8) | 0.40 |
| Diabetes mellitus | 7 (11.7) | 8 (15.4) | 0.56 |
| Current smoking | 21 (35.0) | 20 (38.5) | 0.70 |
| **Clinical exam on admission** | |  |  |
| WFNS grade | 2 [1–4] | 2 [1–4] | 0.31 |
| LOC at ictus | 23 (38.3) | 20 (38.5) | 0.99 |
| **Radiographic findings** | |  |  |
| Modified Fisher grade | 3 [3–3] | 3 [3–4] | 0.54 |
| IVH | 43 (71.7) | 32 (61.5) | 0.26 |
| Hydrocephalus | 35 (58.3) | 27 (51.9) | 0.50 |
| **Therapeutic intervention** | |  |  |
| Clipping | 40 (66.7) | 30 (57.7) | 0.33 |

Variables are presented as mean ± SD, median [interquartile range], or number (%). mRS = modified Rankin scale; WFNS = World Federation of Neurological Surgeons; DCI = delayed cerebral ischemia; LOC = loss of consciousness; IVH = intraventricular hemorrhage. †*p*-values are calculated by Pearson chi-square, Fisher’s exact, or Student's t-test as appropriate.

**Table S3. Multivariable logistic regression analysis to identify predictors of poor clinical outcomes**

|  | Odds ratio (95% CI) | |
| --- | --- | --- |
| Clinical variables | Univariate | Multivariate |
| Age, per 1-year increase | 1.08 (1.02–1.14) | 1.13 (1.04–1.24) |
| Sex, male | 1.77 (0.49-6.40) | 0.43 (0.03-5.66) |
| WFNS grade, per 1-point increase | 2.73 (1.63-4.57) | 2.80 (1.35–5.81) |
| LOC at ictus | 3.97 (1.20-13.22) | 2.29 (0.32-16.62) |
| mFS grade, per 1-point increase | 6.20 (1.41–27.30) | 3.53 (0.35-35.59) |
| Hydrocephalus | 3.96 (0.99-15.85) | 1.14 (0.14–9.43) |

Age, sex, and other variables in the univariate analysis (*p* < 0.10) were included in a multivariable logistic regression analysis.

CI = confidence interval; WFNS = World Federation of Neurosurgical Societies; LOC = loss of consciousness; mFS = modified Fisher Score.

**Cytokine analysis**

Blood samples were collected in K2 EDTA vacutainer tubes and centrifuged within an hour of draw (at 1460 x g for 10 minutes at 4°C) generating plasma. Plasma was centrifuged a second time (at 1460 x g for 10 minutes at 4°C) in order to generate platelet poor plasma. Platelet poor supernatant was collected and stored at -80°C until ready for use. Cytokine sample concentrations were determined using a MAGPIX magnetic bead based ELISA 41-plex assay (Millipore) to test for the following cytokines: the CC chemokines (CCL2, CCL5, CCL7, CCL11 and CCL22), the CX chemokines (CX3CL1 and CXCL1), the macrophage inflammatory proteins (MIP-1α and MIP-1β), the interleukin-1 superfamily (IL-1A, IL-1R1, IL-4, IL-5 and IL-6), the IL-17 family (IL-17a), interleukin-2 (IL-2), interleukin-3 (IL-3), interleukin-7 (IL-7), interleukin-8 (IL-8), interleukin-9 (IL-9), interleukin-10 (IL-10), interleukin-13 (IL-13), interluekin-15 (IL-15), the platelet derived factors (PDGF-AA and PDGF-AB/BB), epidermal growth factor (EGF), fibroblast growth factor-2 (FGF2), colony stimulating factors (CSF2 and CSF3), macrophage-derived chemokine (MDC), tumor necrosis factors (TNF-α and TNF-β), the vascular endothelial growth factors (VEGF-A), IL-12p40, IL-12p70, the soluble CD40-ligand (sCD40L), IL-1β, IP-10, type II interferon family (IFN-γ), interferon-alpha-2 (IFN-α2), the FMS-like tyrosine kinase 3 ligand (FLT-3L) and transforming growth factor alpha (TGF-α). Plasma was analyzed using a multiplex assay, which utilizes fluorescent dye-coated magnetic beads (MAGPIX magnetic bead based ELISA 41-plex assay [Millipore]) and a magnetic bead plate reader (Luminex MagPix [Luminex, Austin, TX]), according to manufacturer's instructions. The plate reader reports results as fluorescence intensity. Mean fluorescence intensity was calculated from the area on the Luminex assay. Per manufacturer's instructions, plasma was diluted 1:100 for measurement of CCL5, PDGF-AA, PDGF-AB/BB and undiluted plasma was used for the 38plex (25ul/well). Unknowns were compared to serial dilutions of manufacturer provided standards and controls. Assays were run in duplicate. In short, samples were incubated in 96 well plates overnight with premixed beads on an agitator at 4°C) and plates were washed. Detection antibodies were added to each well for a one hour incubation period followed by the addition of streptavidin-phycoerythrin and another 30 minutes of incubation. Plates were washed and the beads were re-suspended in drive fluid and the plate was run on the MAGPIX with xPONENT software. All cytokine values were expressed in pictograms per milliliter (pg/ml).

**Infection:**

To investigate the effect of infection, we examined the number of subjects in our study population who had any type of culture proven infection. We used culture positive infection as SAH subjects develop fever and elevated white count because of inflammation and not necessarily infection. We also differentiated between types of infection and recorded the time elapsed since the subject was admitted to the time when an infection was suspected and a sample was sent for culture. In the study cohort, 17 subjects developed an infection from T_1_ to T_4_ that was confirmed from the culture of either blood, cerebrospinal fluid, sputum, urine or bronchoalveolar lavage during the study period. 2(3.3 %) of the subjects at T_1_, 2(3.3 %) of the subjects at T_2_, 4+1(8.3 %) at T_3_ and 8 (13 %) of the subjects at T_4_ had any type of infection. 8(13%) of the subjects developed infection after T_4_ period. There are no differences in the infection status of patients across the DCI vs no-DCI group and across good and poor outcomes as well. Our findings that a) the interaction between CCs are highest at T_2_ and numbers of subjects with infection at T_2_ versus T_3_ and T_4_ suggests that b) the observed elevations in cytokines and the interactions are likely not from infection. We can only say this with modest confidence as the presence and absence of active infection is difficult state definitively. It is possible that subjects at T_1_ and T_2_ had active **
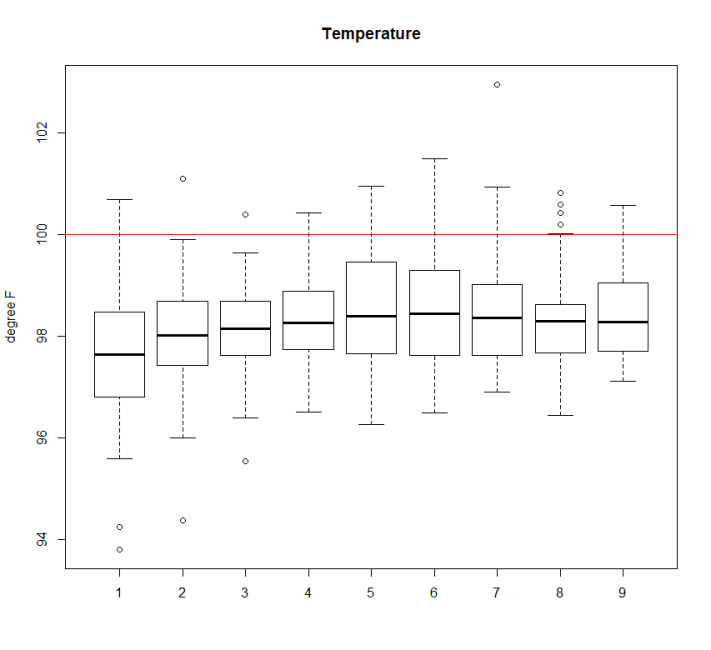
**infections but they were not identified. This is a common issue in any study examining inflammation after acute injuries. Furthermore, we examined the average values of temperature each day, from admission till day 8 in our study population (Supplemental Figure 3). Temperature was the lowest at the first two days after admission (which coincides with the periods T1 and T2). Since only about 2% of the subjects had any infection at T1 and 8% at T2, it is likely that these values are reflective of the underlying pathological process rather than the infection. However, temperature is high at around T4. These findings suggest that temperature rise be a secondary reaction to infection suggesting that infections were likely the result of subjects being in the ICU/hospital longer from neurologic complications. This points some of the novelty of our findings. We found that in the set of cytokines that we examined, the interactions were highest early on, making it at least temporally a potential cause of DCI.

**Supplemental Figure 3:** The average values of temperature for the study cohort for day of admission to day 9. (Red line drawn at 100 Fahrenheit)
